# Generalized Joint Hypermobility: A Statistical Analysis Identifies Non-Axial Involvement in Most Cases

**DOI:** 10.3390/children11030344

**Published:** 2024-03-14

**Authors:** Mateus Marino Lamari, Neuseli Marino Lamari, Michael Peres de Medeiros, Matheus Gomes Giacomini, Adriana Barbosa Santos, Gerardo Maria de Araújo Filho, Eny Maria Goloni-Bertollo, Érika Cristina Pavarino

**Affiliations:** 1 Department of Epidemiology and Public Health, Medical School of São José do Rio Preto (FAMERP), Av. Brigadeiro Faria Lima, 5416, Vila São Pedro, São José do Rio Preto 15090-000, SP, Brazil; mateus.lamari@edu.famerp.br; 2 Department of Neurological Sciences, Psychiatry and Medical Psychology, Medical School of São José do Rio Preto (FAMERP), Av. Brigadeiro Faria Lima, 5416, Vila São Pedro, São José do Rio Preto 15090-000, SP, Brazil; michael.medeiros@edu.famerp.br (M.P.d.M.); gerardo.filho@famerp.br (G.M.d.A.F.); 3 Foundation of the Regional Medical School of São José do Rio Preto (FUNFARME), Av. Brigadeiro Faria Lima, 5544, Vila São Pedro, São José do Rio Preto 15090-000, SP, Brazil; matheus.giacomini@edu.famerp.br; 4 Department of Computer Science and Statistics, São Paulo State University (UNESP), R. Cristóvão Colombo, 2265, Jardim Nazareth, São José do Rio Preto 15054-000, SP, Brazil; adriana.barbosa@unesp.br; 5 Genetics and Molecular Biology Research Unit, Department of Molecular Biology, Medical School of São José do Rio Preto (FAMERP), Av. Brigadeiro Faria Lima, 5416, Vila São Pedro, São José do Rio Preto 15090-000, SP, Brazil; eny.goloni@famerp.br (E.M.G.-B.); erika@famerp.br (É.C.P.)

**Keywords:** child, pediatrics, joint hypermobility, hypermobility spectrum disorders, Ehlers–Danlos syndrome

## Abstract

Context: Joint hypermobility (JH) represents the extreme of the normal range of motion or a condition for a group of genetically determined connective tissue disorders. Generalized joint hypermobility (GJH) is suspected when present in all four limbs and the axial skeleton, scored in prepubescent children and adolescents by a Beighton Score (BS) ≥ 6. Parameters are also used to identify GJH in hypermobile Ehlers–Danlos syndrome (hEDS) and hypermobility spectrum disorders (HSDs). The purpose of this study is to characterize children with JH based on the location of variables in the BS ≥ 6 and identify children with JH in the axial skeleton, upper limbs (ULs), and lower limbs (LLs) simultaneously. Methods: We analyzed 124 medical records of one- to nine-year-old children with JH by BS. Results: The characterization of GJH by combinations of the axial skeleton, ULs, and LLs simultaneously totaled 25.7%. BS = 6 and BS = 8 consisted of variables located in ULs and LLs. BS = 7 included the axial skeleton, ULs, and LLs. BS ≥ 6 represents the majority of the sample and predominantly girls. Conclusions: BS ≥ 6 represents the majority of the sample and predominantly girls. Most characterized children with GJH present BS = 6 and BS = 8 with variables located only in ULs and LLs, a condition that does not imply the feature is generalized. In children, BS = 7 and BS = 9 characterize GJH by including the axial skeleton, ULs, and LLs. These results draw attention to the implications for defining the diagnosis of hEDS and HSDs.

## 1. Introduction 

Joint hypermobility (JH) can be the extreme of the normal spectrum of range of motion or a condition for a group of hereditary connective tissue disorders. The condition is influenced by age, sex, and ethnicity, with a Gaussian distribution in the spectrum of the physiological range of motion and is also considered a genetically determined deviation from normality [1,2]. JH is a common characteristic in humans that may be associated with musculoskeletal complaints and is considered a diagnostic criterion for hypermobile Ehlers–Danlos syndrome (hEDS) and hypermobility spectrum disorders (HSDs), both of which are common in the general population, with extreme clinical variability and an uncertain ethiopathogenesis [3]. Associations have also been reported between generalized joint hypermobility (GJH) and neurodevelopment as well as psychopathology [4]. 

One should bear in mind that JH is a descriptor and not a diagnosis [5]. It is often a characteristic of a larger, rarely diagnosed syndrome [6] and erroneously considered a harmless ability by most healthcare providers [7]. The GJH is defined as JH in all four limbs and the axial skeleton and can occur in families with distinct genetic inheritance patterns, with a predominance of the autosomal dominant pattern [5], likely related to genes that code collagen or a collagen-modifying enzyme [8,9]. Connective tissue frailty is identified in tendons, fascia, septa, ligaments, and joint capsules due to the structural deficiency of proteins [10]. 

Studies published prior to 2017 reported that the prevalence of GJH varied with age, sex, and ethnicity, with rates ranging from 2 to 64.6% in different populations [11,12]. GJH is common in childhood and is found in 8 to 39% of schoolchildren [13], 41% of children and adolescents [14], and 64.6% of preschool children [11]. The condition is more frequent in women and children [15,16] and individuals of African descent [17]. The high frequency of JH in children [18] makes it difficult to distinguish those with a normal physical characteristic from those with an underlying disease [19].

Beighton, Solomon, and Soskolne [17] proposed a method that attributes individuals weighted scores for each region of the body, with the Beighton Score (BS) ranging from zero to nine points for the characterization of GJH. This has been the most widely used method in the specialized literature in the last 50 years. The BS was created for the purpose of screening in epidemiological studies and not for clinical use. The method is fast and easy to execute, but is strongly biased toward the ULs [20]. In 2005, Lamari et al. [11] pointed out the need for specific criteria for children. Tofts et al. [21] recently reported the need for specific pediatric criteria and proposed a diagnostic structure for JH in children.

In 2017, an international consortium for EDS proposed the international classification in 13 subtypes with diagnostic criteria for each one [2]. hEDS is among the 13 subtypes, similar to HSDs [5]. The consortium [2] defined BS ≥ 6 for identifying the occurrence of GJH in children and prepubescent adolescents. 

Comorbidities described in hEDS/HSDs are also found in the pediatric population [20,21,22]. Lamari and Beighton [22] illustrated sequelae in hypermobile children, pre-adolescents, and adolescents located in the chest, feet, and spinal column and related to oral health. 

The characteristic of JH in HSDs may be generalized, localized, peripheral, or historical [5]. The diagnosis of hEDS/HSDs is clinical and based on widely accepted diagnostic criteria [2], which include the characteristics of GJH. 

The purpose of this study is to characterize children one to nine years of age with JH using the BS; identify variables based on body location that compose the BS ≥ 6 score; investigate the possible influence of UL variables on the composition of BS ≥ 6; identify children with BS ≥ 6 with the inclusion of the ULs, LLs, and axial skeleton concomitantly; investigate the influence of sex on the variables of the BS and of the BS; and investigate whether the BS ≥ 6 parameter alone (without considering the composition of its variables) is sufficient for defining GJH. 

## 2. Methods

This study received approval from the Human Research Ethics Committee of the São José do Rio Preto School of Medicine (FAMERP) (approval certificate number: 36145820.6.000.5415) in accordance with regulatory norms stipulated in Resolution 466/2012 of the Brazilian National Board of Health. 

An observational, quantitative, retrospective, cross-sectional study was conducted with the analysis of the clinical records with JH from the Lamari Physiotherapy Clinic, Ltd. in the city of São José do Rio Preto, SP, Brazil. The inclusion criterion was patient records with information of interest for the data-collection instrument. 

Data were analyzed from the records of 124 children from five states of Brazil treated in the period from 2014 to 2020. The sample comprised children one to nine years of age [mean age: 5.5 ± 2.4 years; 74 girls (59.7%) and 50 (40.3%) boys]. Mean and median age of the girls was seven years, with a mode of 8 ± 1.81 years. For the boys, mean age was six years, with a median of seven years and mode of 8 ± 2.24 years. 

### 2.1. Data-Collection Instrument 

An instrument was designed by the researchers for recording the data collected. Data were collected from the records of patients with JH referring to the period from January 2014 to March 2020, considering the results of general and specific physical examinations as well as characteristics associated with JH, a family history of JH, and the BS [17]. 

### 2.2. Analysis of GJH Based on BS 

**Figure 1:**
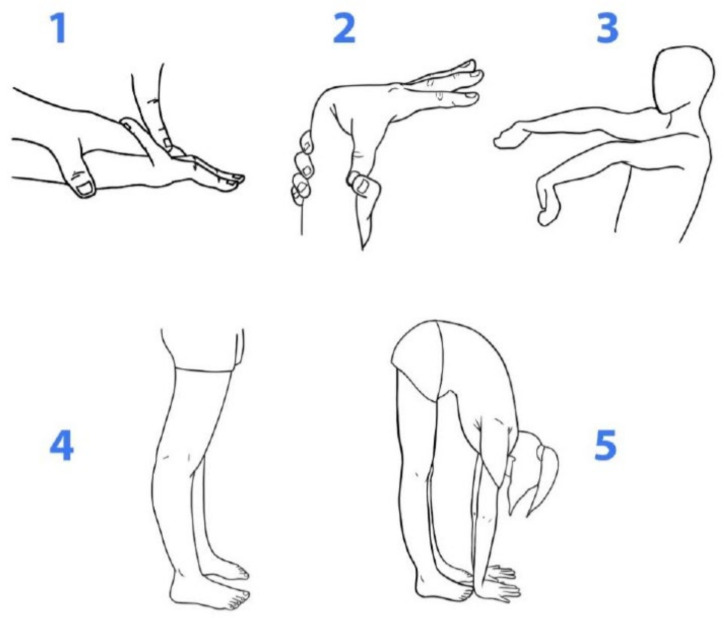
Illustration of five variables for assessment of GJH: 1. 5th finger hyperextension is defined by passive extension with an angle > 90°; 2. wrist hyperflexion is defined by passive thumb touch to the forearm flexor region; 3. elbow hyperextension is defined by active extension with an angle > 10°; 4. knee hyperextension is defined by active extension with an angle > 10°; 5. anterior spinal column hyperflexion is defined by palms touching the ground with extended lower limbs.

Joint mobility was investigated in five regions of the body for the determination of the BS. One point was attributed for each positive result on each side. The BS was obtained by the sum of the points and ranged from 0 to 9. The following aspects were investigated: passive extension of the fifth finger (F), passive apposition of the thumb on the forearm (A), active hyperextension of the elbow (E) and knee (K) and anterior trunk flexion (ATF). The values were obtained with the aid of a goniometer, with the exception of the spinal column variable (Figure 1).

### 2.3. Statistical Analysis

Data analysis involved descriptive statistics; tables of unidimensional and bidimensional frequencies for categorical variables; the Fisher’s exact test to evaluate the statistical significance of sex in the contingency tables; Mann–Whitney U test, for comparing independent samples; and basic graphs to represent percentage distributions. The significance level was set at 5% (*p* < 0.05). The Minitab v.16 software was used for the calculations [23,24].

## 3. Results

### 3.1. Characteristics of JH Considering Beighton Variables and Distribution by Sex

**Figure 2:**
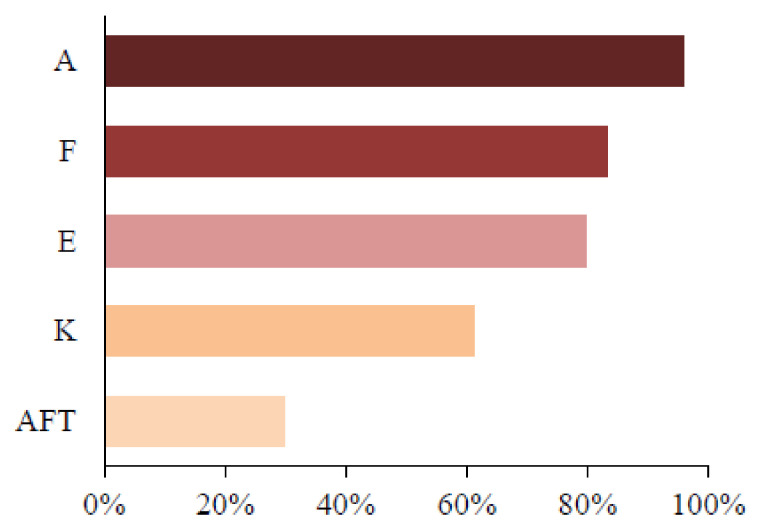
Percentage distribution of Beighton variables A, F, E, K, and ATF in overall sample.

As shown in Figure 2, A was the most frequent Beighton variable in the overall sample (n = 119; 96%), followed by F (n = 103; 83.1%), E (n = 99; 79.8%) and K (n = 76; 61.3%). ATF was the least frequent variable (n = 37; 29.8%). 

The combination of body regions with JH was characterized in the sample, as shown in Table 1. The sample was subdivided so that the variables were grouped in different regions with results for each location. Combinations of upper- and lower-limb variables were found in 39.5% of the patients; combinations only involving the ULs were found in 28.2%; and combinations that included ATF and both the ULs and LLs were found in 25.7%. These findings suggest that 25.7% of the children had GJH, as the characteristic was present in the axial skeleton and limbs. Considering the occurrence of BS = 6 and BS = 8 in the overall sample, these scores are composed of variables located exclusively in the ULs or in both the upper and LLs, with the absence of the ATF variable. Therefore, only children with a BS = 7 and a BS = 9 exhibited GJH.

Table 2 displays the results related to the descriptor variables of JH in the overall sample and stratified by subsamples. A statistically significant difference between girls and boys was only found for the ATF variable (*p* = 0.026, Fisher’s exact). Sex exerted no influence on the other variables, as the proportions of JH were statistically similar between girls and boys. 

**Figure 3:**
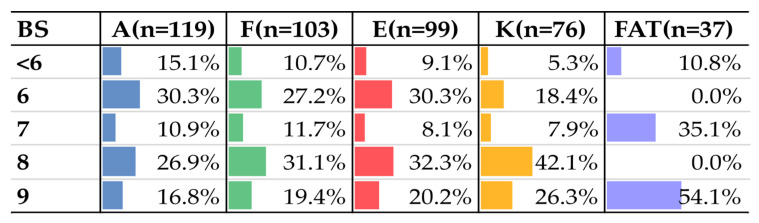
Absolute and percentage distribution BS and variables.

Figure 3 displays the important associations between BS and each variable. Among the 119 children with JH of the wrist, demonstrated by the apposition of the thumb (A), 30.3% had BS = 6. The children with JH in the form of the hyperextension of the fifth finger (n = 103), elbow (n = 99), and knee (n = 76) had greater frequencies of BS = 8, reaching 31.1%, 32.3% and 42.1% for each Beighton variable, respectively. Among the 37 children with JH in the form of ATF, 54.1% had BS = 9, denoting GJH (axial skeleton, ULs, and LLs).

### 3.2. Characteristics of GJH Based on BS and Distribution by Sex 

The results of the analysis of the overall sample that score and characterize current JH in five body regions with the BS ranging from zero to nine points are detailed with the respective frequency of the occurrence of each value in Table 3, which includes the BS of the overall sample and is stratified by sex. The numbers in bold type correspond to patients with BS ≥ 6, who totaled 81.4%. A BS = 6 was more frequent among boys (38%), whereas a BS = 8 was more frequent among girls (27%), suggesting that GJH is more common in the female sex. 

As shown in Table 4, BS ranges were defined for the regrouping of the participants and to enable the comparison of proportions between the sexes using Fisher’s exact test. Among those with BS ≥ 6, 62 (83.8%) were girls and 39 (78%) were boys. However, the difference was not statistically significant (*p* = 0.483). For BS ≥ 7, 45 participants (60.8%) were girls and 20 (40.0%) were boys, with a statistically significant difference between sexes (*p* = 0.028). Both BS ≥ 8 and BS = 9 were more frequent among the girls, but the differences between sexes were nonsignificant (*p* = 0.194 and *p* = 0.144, respectively). Therefore, the influence of sex was evident in the BS ≥ 7 range, but was not found in the other BS ranges. Considering the overall sample, the nonparametric Mann–Whitney test revealed a significant difference in BSs between the sexes (*p* = 0.046). 

## 4. Discussion 

The present study characterized children with JH based on the Beighton variables, the location of these variables in the body, and the combination of variables that result in a BS in both the overall sample and stratified by sex in a narrow age range (one to nine years). This strategy could contribute to avoiding the burden of a late diagnosis as well as morbidity and disability due to the ineffective treatment of JH, as recently highlighted in the paper by Walter, Dai Z. and Wang [25]. Castori [3] also highlights the need for a better understanding of relationships among current criteria, which could contribute to the definition of more balanced criteria in the future.

The condition of GJH is defined by the BS [17], which is widely used for the assessment of GJH [26] and was modified in 2017 [2]. This method has been the most widely used tool for this purpose in the specialized literature. Adaptations of the BS were recently proposed considering different age groups [2], with BS ≥ 6 defined for children and prepubescent adolescents. In the same year, Castori et al. [5] proposed criteria for HSDs with the identification of JH per location in the affected region of the body in the present as well as including past occurrence of the characteristic. 

The present study found a predominance of JH in the ULs in the overall sample, with the majority of children of both sexes exhibiting BS ≥ 6. Similar results were identified in 482 patients with JH in a previous study [16], in which JH also predominated in the ULs, with 64.73% of the sample obtaining BS ≥ 6. The analyses with combinations of all variables of the method used in the present study revealed that BS = 6 may correspond to variables of the ULs alone or both the ULs and LLs, excluding the axial skeleton. Similar results were reported in a study involving 1120 children from four to seven years of age, 26.9% of whom had BS ≥ 6 [11,27]. In a study involving prepubescent children, Yazgan at al. [28] investigated the prevalence and characteristics of GJH defined by a BS ≥ 6, which was found in 13.3% of the sample. The difference in the age range of the studies by Lamari, Chueire and Cordeiro [11] and Yazgan et al. [28] may be explained by the particularities of each study, with children in the preschool and prepubescent age ranges, respectively. It is noteworthy that the present study analyzed the sample considering the new parameters of the BS [2]. 

As GJH is defined as the presence of JH identified in all four limbs and the axial skeleton concomitantly [5], there is a need to rethink the criteria for characterizing the generalized characteristics. The present study showed that a BS = 6 and a BS = 8 correspond to the localized form, without the inclusion of ATF. In this context, a BS = 5 may also be suggestive of the inclusion of both the axial skeleton and appendicular skeleton in children, which can exert an impact on the diagnosis, treatment, and the results of studies. Considering that the Committee on Behalf of the International Consortium on the Ehlers–Danlos Syndromes proposes a BS ≥ 5 for pubertal men and women up to the age of 50, and a BS ≥ 4 for those > 50 years of age for hEDS [2], suggestively, in the logic of the analyses of this study, it is expected that the same interpretation will apply to older age groups, with the axial axis potentially not being included. More recently, a BS ≥ 6 was maintained for the characterization of pediatric GJH and pediatric HSDs [21]. 

Our results suggest that other studies should be conducted with these purposes, as the same is expected to occur in adolescents and adults, but with a smaller number of variables due to the reduction in joint mobility expected with the increase in age, as the BS in other age groups is predominantly a BS ≥ 4 and a BS ≥ 5, corresponding more to localized JH. These results call attention to the epidemiological implications, as the BS is part of the main diagnostic criteria for hEDS and HSDs, and also has implications for clinical practice and research. One should bear in mind that HSDs and hEDS are considered common in the general population [3]. 

With the purpose of gaining a better understanding of GJH based on the Beighton variables, the present study identified thumb apposition (A) as the most frequent variable, followed by the hyperextension of the fifth finger (F), hyperextension of the elbow (E), and hyperextension of the knee (J). ATF was the least frequent variable. Similar results were found in a study involving 1120 male and female Brazilian preschool children [11]. In the overall sample, BS ≥ 6 was found in 26.9%, with lower frequencies for the variables ATF and knee hyperextension, as found in the present investigation. 

In 2005, Lamari et al. [11] concluded that the parameter also used for children needed to be reviewed and proposed that other studies could have compromised results for having included different ages in the same sample and analyzed with the same criteria. Adjustments of the BS were considered and occurred 18 years later [2].

The quantification of individuals with a given combination of body regions with JH is another aspect that characterizes the sample in the present study. Thus, the sample was subdivided, grouping those with JH in different regions. The results showed that combinations involving A, F, E, and J (without the ATF variable) were more prevalent.

The analysis of the descriptor variables of JH in the overall sample and stratified by subsamples revealed a statistically significant difference between sexes only for ATF, which was more frequent among the girls. No significant difference between sexes was found in the analysis of a BS ≥ 6, whereas a statistically significant difference was found for a BS ≥ 7, which was more frequent among the girls. Therefore, the present study makes relevant contributions to reflect upon with regard to sex. The odd score suggests the possibility that ATF produced the difference for the female sex, which is in agreement with the results of the analyses of the individual variables for the girls. The study by Lamari et al. [11] involving 1120 preschool children showed that ATF was the least frequent variable in the overall sample and was more prevalent in the female sex. Recent publications describe data in agreement with the present results [16,21,22], also reporting a greater frequency in the female sex. 

Throughout early childhood, children with JH in preclinical stages may exhibit perceptible musculoskeletal characteristics related to JH during the tissue-maturation process [11,20,22]. In the present study, children with hypermobility had predominantly high BSs. The incorporation of a rapid assessment of JH in the routine of pediatric appointments could lead to early detection as well as the prevention of deformities, incapacities, and even physical disabilities [22,27]. Thus, analysis involving the clinical history, inspection, physical examination, and special tests can reveal the characteristics of JH [16]. The rapid test employed in the present study involving the BS was created for epidemiological assessments for the purposes of screening and not for clinical use [17,20]. Early screening can help guide treatment based on the evolution period and severity of the condition, with the implementation of early interventions [29]. 

The 2017 diagnostic criteria for hEDS [2] and HSDs [5] were established based on the consensus of specialists and studies involving adults, which are difficult to use on children and biologically immature adolescents who have not yet developed a stable phenotype. Thus, the Pediatric Working Group of the International Consortium on EDS and HSD developed a framework for pediatric diagnoses [21] based on the review of evidence, as the criteria established for hEDS [2] are reserved for biologically mature adolescents and adults. Efforts are still needed for a better identification of the characteristics of GJH in different age groups and to contribute to physiotherapeutic care with better guidance for health promotion in this population, the prevention of mechanical impacts and deformities in affected joints, as well as a better understanding of the semiology in clinical stages of manifestation related to hypermobility in other age groups. 

Another aspect that merits attention is the prevalence of GJH, which ranges from 2 to 64.6% in different populations [11,12,16,18,30,31,32], which further hampers the understanding of the prevalence in population-based studies, except for children and the female sex, for which more information is available in the literature [11,13,16,33]. The present study was conducted with male and female children with hypermobility, and the sample was composed predominantly of girls. Castori et al. [34,35] and Lamari et al. [16] suggest that the greater frequency in the female sex is associated with the composition of muscle mass and ligament rigidity, with greater joint stability in the male sex. The authors also point out that the female sex is more likely to seek early medical assistance. The results of the present study are in line with the specialized literature, as JH was more prevalent in the female sex even in a narrow age range. The analyses of the individual scores and BSs revealed that the female sex only exerted an influence on the ATF variable. 

These conditions justify the present study, as a small number of studies have been conducted involving children of both sexes in a narrow age range. This justification is strengthened by our observations in daily clinical practice for approximately 40 years with individuals with JH in different age ranges, including children. A better understanding of the characteristic of GJH in children is expected to enable effective interventions in other age groups. There is a need for studies involving both sexes and analyses by age group for the identification of variables per region of the body and the establishment of BS to ensure the diagnosis, establishment of treatments, and reliable results of studies. 

## 5. Conclusions

The analysis of the variables that compose the Beighton for scoring JH in children revealed that thumb apposition to the forearm is the most frequent variable, followed by the hyperextension of the fifth finger, elbow, and knee. The ATF variable is the least frequent in both sexes and is found significantly more often in girls. The analysis also reveals that most children with hypermobility have BS = 6 and BS = 8, with variables located in the limbs, but not the trunk, which does not enable characterizing JH as generalized. The sum of the Beighton variables in these cases is insufficient for the characterization of GJH. In contrast, a BS ≥ 7 and a BS ≥ 9 include the axial skeleton and all four limbs, which denotes the condition of GJH. GJH should only be considered when the characteristic is present in all four limbs and the axial skeleton. The BS ≥ 7 category is significantly influenced by sex, found predominantly in girls as a function of ATF. These results suggest the need for further studies with different age ranges and both sexes for the identification of the variables that constitute the BS to ensure a proper diagnosis, effective treatments, and reliable results of studies. The results also call attention to the implications for the definition of the diagnosis of hEDS and HSDs as well as implications for clinical practice and research. Studies with this purpose should be conducted in different countries, considering the peculiarities of racial miscegenation of the Brazilian population and the influence of ethnicity on joint mobility.

## Figures and Tables

**Table 1:** Absolute and percentage distribution of combinations of Beighton variables A, F, E, K, and ATF in ULs, LLs, and axial skeleton in overall sample.

Combination	Frequency (%)	Location
A, F, E, K	33 (26.6%)	ULs/LLs
A, E, K	8 (6.5%)	Uls/LLs
A, F, K	6 (4.8%)	Uls/LLs
E, K	2 (1.6%)	Uls/LLs
A, F, E	21 (16.9%)	Uls
A, F	10 (8.1%)	Uls
A, E	4 (3.2%)	Uls
A, F, E, K, ATF	20 (16.1%)	Uls/LLs/Axial skeleton
A, F, E, ATF	7 (5.6%)	Uls/Axial skeleton
A, F, K, ATF	5 (4.0%)	Uls/LLs/Axial skeleton
Others	7 (5.6%)	-
Total	124 (100%)	

**Table 2:** Absolute and percentage frequencies of Beighton variables stratified by sex and corresponding *p*-values.

Beighton Variable	Hypermobility Present		*p*-Value (Fisher’s Exact Test)
	Female	Male	
F	60 (81.1%)	43 (86.0%)	0.626
A	71 (95.9%)	48 (96.0%)	0.999
E	62 (83.8%)	37 (74.0%)	0.254
K	50 (67.6%)	26 (52.0%)	0.093
ATF	28 (37.8%)	09 (18.0%)	0.026

**Table 3:** Absolute and percentage distribution of BS in overall sample and stratified by sex.

BS	Overall	Female	Male
2	2 (1.6%)	1 (1.4%)	1 (2.0%)
3	2 (1.6%)	1 (1.4%)	1 (2.0%)
4	17 (13.7%)	8 (10.8%)	9 (18.0%)
5	2 (1.6%)	2 (2.7%)	0
6	36 (29.0%)	17 (23.0%)	19 (38.0%)
7	13 (10.5%)	10 (13.5%)	3 (6.0%)
8	32 (25.8%)	20 (27.0%)	12 (24.0%)
9	20 (16.1%)	15 (20.3%)	5 (10.0%)
Total	124	74	50

**Table 4:** Absolute and percentage distribution of BS ranges stratified by sex and corresponding *p*-values.

BS Range	Female	Male	*p*-Value (Fisher’s Exact Test)
≥6	62 (83.8%)	39 (78%)	0.483
≥7	45 (60.8%)	20 (40%)	0.028
≥8	35 (47.3%)	17 (34%)	0.194
9	15 (20.3%)	05 (10%)	0.144

## Data Availability

All these data are not publicly available due to privacy and ethical restrictions. The data were collected from a patient database that is not publicly available.
